# Particle Tracking Facilitates Real Time Capable Motion Correction in 2D or 3D Two-Photon Imaging of Neuronal Activity

**DOI:** 10.3389/fncir.2017.00056

**Published:** 2017-08-15

**Authors:** Samira Aghayee, Daniel E. Winkowski, Zachary Bowen, Erin E. Marshall, Matt J. Harrington, Patrick O. Kanold, Wolfgang Losert

**Affiliations:** ^1^Department of Physics, University of Maryland College Park, MD, United States; ^2^Department of Biology, University of Maryland College Park, MD, United States

**Keywords:** motion correction, mesoscale neuroscience, calcium imaging, image registration, TPSM data registration

## Abstract

The application of 2-photon laser scanning microscopy (TPLSM) techniques to measure the dynamics of cellular calcium signals in populations of neurons is an extremely powerful technique for characterizing neural activity within the central nervous system. The use of TPLSM on awake and behaving subjects promises new insights into how neural circuit elements cooperatively interact to form sensory perceptions and generate behavior. A major challenge in imaging such preparations is unavoidable animal and tissue movement, which leads to shifts in the imaging location (jitter). The presence of image motion can lead to artifacts, especially since quantification of TPLSM images involves analysis of fluctuations in fluorescence intensities for each neuron, determined from small regions of interest (ROIs). Here, we validate a new motion correction approach to compensate for motion of TPLSM images in the superficial layers of auditory cortex of awake mice. We use a nominally uniform fluorescent signal as a secondary signal to complement the dynamic signals from genetically encoded calcium indicators. We tested motion correction for single plane time lapse imaging as well as multiplane (i.e., volume) time lapse imaging of cortical tissue. Our procedure of motion correction relies on locating the brightest neurons and tracking their positions over time using established techniques of particle finding and tracking. We show that our tracking based approach provides subpixel resolution without compromising speed. Unlike most established methods, our algorithm also captures deformations of the field of view and thus can compensate e.g., for rotations. Object tracking based motion correction thus offers an alternative approach for motion correction, one that is well suited for real time spike inference analysis and feedback control, and for correcting for tissue distortions.

## Background and introduction

Behaviorally relevant information in the brain does not reside in the firing events of individual neurons, but instead in the collective activity of groups of neurons. Thus, understanding the collective behavior of neurons is essential for understanding how the brain processes information and encodes memory. Two-photon laser scanning microscopy (TPLSM) of neuronal activity using Ca^2+^ indicators is a powerful approach that allows for analysis of the inner workings of the brain at the level of single cells for large groups of neurons within an awake behaving organism (O'Connor et al., 2009; Peron S. et al., 2015). While electrophysiology approaches allow for very accurate measurements of the neuronal circuit a few neurons at a time, TPLSM allows for the simultaneous observation of hundreds to thousands of neurons (Peron S. P. et al., 2015) and thus yields information on the collective behavior of groups of neurons.

Measurements of collective dynamics require accurate detection of neuronal activity of tens to thousands of individual neurons. Since detection errors grow exponentially with the size of the observed neuronal population, detection inaccuracies would lead to spurious measurements and conclusions. For example, with 97% accuracy for identification of a single neuronal spike one can only achieve 74% accuracy for identifying simultaneous activity of 10 neurons. Thus, to detect synchronous activity of 100 neurons with more than 95% confidence, the detection of single neuron events must be made with close to 100% accuracy.

Precise spike inference is particularly challenging in two-photon Ca^2+^ imaging data where neuronal activity is inferred by brightness fluctuations of imaged pixels within the regions of interest (ROI). Current indicators, such as GCaMP6 (Chen et al., 2013) are expressed in the cytoplasm of the neuron, forming a ring surrounding the nucleus, thus when imaging a large population of neurons, relatively few pixels contribute to the cellular signal. Displacement of the neuronal somata over time from the defined ROIs can therefore perturb the inferred activity. Therefore, before extracting neuronal activity from the image plane, it is essential to accurately correct for motion which can be particularly large in awake behaving preparations. A number of motion correction algorithms have been developed and adopted over the past two decades. For example, a line by line motion correction method based on Hidden Markov Model (HMM) was introduced to correct for line to line jitter notable at slower scanning rates (Dombeck et al., 2007). Other algorithms have been used for correction of both slow and fast movements (Greenberg and Kerr, 2009). However, the line to line jitter became less relevant at higher frame rates and frame-to-frame motion correction is the main challenge today. One solution is a cross-correlation based algorithm to find the translational shift of each frame with a reference image (Miri et al., 2011). TurboReg (Thevenaz et al., 1998) is a commonly used implementation of this approach. A faster and more accurate implementation called MoCo has recently been developed for Fiji Toolbox (Dubbs et al., 2016). Fourier transform based methods, e.g., the DFT approach (Guizar-Sicairos et al., 2008) are fast, but do not detect rotation. However, imaging ever-increasing fields of view to capture the activity of millions of neurons (Sofroniew et al., 2016) will require methods that go beyond rigid body translations and include rotations and also deformations in the field of view. Moreover, to facilitate closed loop experiments, motion correction has to operate fast enough to extract neuronal activity in real time. Two recent preprints (Pachitariu et al., 2016; Pnevmatikakis and Giovannucci, 2017) indicate complementary ongoing studies using a block-wise phase-correlation algorithm. The preprints highlight either real time capability or the ability to detect deformations, but not both. Here we introduce a particle-tracking based approach that is fast enough for real time implementation yet suitable for detection of large jitter and of image rotations and can be simply expanded to correct for distortions.

We have developed a suite of analytical tools to quantify brain motion that adapts sub-pixel accuracy object-tracking tools from the field of soft-matter physics. We use these tools to characterize brain motion and to compensate for it with sub-pixel resolution at real time speed. In addition, we compare the performance of our tools to established algorithms that are used widely for motion correction. Specifically, we introduce a particle-tracking based image analysis pipeline capable of significantly reducing in-plane motion, which yields more accurate spike inference measurements. Though we only tested the algorithm off-line, the algorithm is fast enough (processing up to 85 frames per second) to be used in real time analysis of calcium transients, and is amenable to extensions for 3D motion correction.

## Methods

### Animals

All procedures were approved by the University of Maryland Institutional Animal Care and Use Committee.

Adult wild type (C57) mice (>P40, range P40–P100 at the time of experiments) of both genders underwent a single aseptic surgical procedure in which they received an implant of a titanium headplate, intracortical injections of adeno-associated virus delivering the gene of a genetically encoded calcium indicator, and chronic cranial windows (Goldey et al., 2014). The titanium headplate design was a modified version of headplate presented in Guo et al. (2014) to allow access to auditory cortex. Adeno-associated virus AAV1.hSyn1.mRuby2.GSG.P2A.GCaMP6s.WPRE.SV40 (Addgene50942) (Rose et al., 2016) was obtained from UPenn Vector Core and injected into several locations (~30 nL per site; ~300–350 μm from pial surface; 3–6 sites) in the auditory cortex using a Nanoject system (Drummond). This sequence allows for the expression of mRuby2 throughout the neuron as well as expression of Gcamp6s. The craniotomy was sealed with a chronic cranial window (Goldey et al., 2014). The entire implant except for the imaging window was then coated with black dental cement created by mixing standard white powder (Dentsply) with iron oxide powder (AlphaChemical, 3:1 ratio) (Goldey et al., 2014). At the conclusion of the procedure, the animals were given a subcutaneous injection of meloxicam as an analgesic. After complete recovery from surgery (>1–2 weeks), animals were habituated to the restraint system over several sessions. After 1 week of habituation, 2-photon imaging experiments commenced.

### Imaging

For 2-photon imaging, we used a scanning microscope (Bergamo II series, B248, Thorlabs) coupled to a pulsed femtosecond Ti:Sapphire 2-photon laser with group velocity dispersion compensation (Vision S, Coherent). The microscope was controlled by ThorImageLS software. The laser was tuned to λ = 940 nm in order to simultaneously excite GCaMP6s and mRuby2. Red and green signals were collected through a 16 × 0.8 NA microscope objective (Nikon). Emitted photons were directed through 525/50-25 (green) and 607/70-25 (red) band pass filters onto cooled GaAsP photomultiplier tubes (Hammamtsu). The field of view was 370 × 370 μm. Imaging frames of 512 × 512 pixels (pixel size 0.72 μm) were acquired at 30 Hz by bidirectional scanning of an 8 KHz resonant scanner. Beam turnarounds at the edges of the image were blanked with a Pockels Cell. The average power for imaging was <70 mW, measured at the sample. For single plane imaging, imaging sites were ~150–270 μm from the pia surface.

For volume scanning, axial motion was controlled with a piezo collar (Physik Instrument) attached to the microscope body. The microscope objective was moved smoothly through a z-distance of ~60 μm across 13 imaging planes, two frames as the objective returned to the starting position (i.e., flyback frames) were discarded. All images were acquired at 352 × 352 pixels (253 μm × 253 μm). The inclusion of multiple z-planes reduced our temporal resolution to ~3 frames per second.

### Motion correction algorithm

We use a cell tracking approach for motion correction, which consists of three steps: cell extraction, tracking cells and calculating offsets and rotations of each frame, and correcting for it.

#### Cell finding algorithm

**Figure 1:**
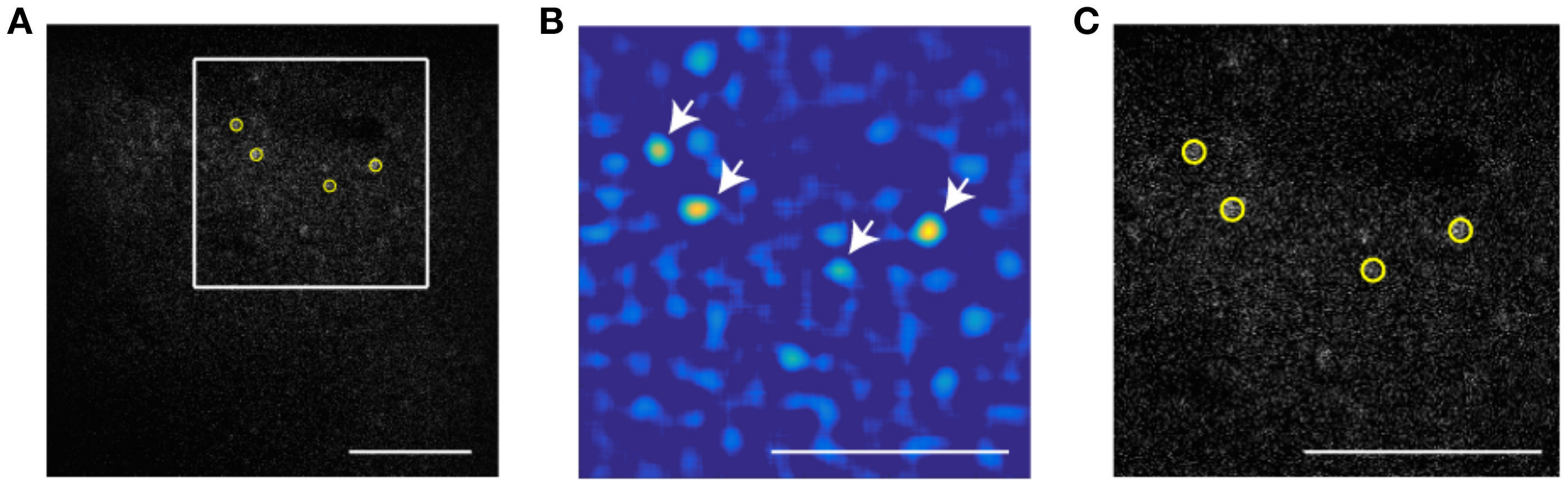
Particle finding via bandpass filtering in the static channel. The scale bar is 100 μm. **(A)** Typical frame acquired by TPSM. The low signal to noise ratio is in most cases unavoidable due to high speed scanning. **(B)** Bandpassed image of the same frame with lower and higher bandpass of, respectively 4 and 13 pixels. **(C)** The four brightest cells extracted by the algorithm (yellow circles) Scale bar is 100 microns. White arrows show the brightest cells in the bandpassed image.

To extract the position of cells in the invariant channel over background we use bandpass filtering and peak finding. Figure 1A shows a sample image of a neuronal population with low signal to noise ratio (SNR). In this example, we use a 16x objective and image 512 × 512 pixels (at 0.72 μm per pixel) such that a cell diameter is about 15 pixels. To enhance the SNR, the image is bandpassed with a lower threshold of 4 pixels to remove pixel scale noise and an upper threshold comparable to (typically slightly smaller than) the cell size (13 pixels). The resulting image is shown as Figure 1B. On this filtered image we use automatic peak finding routines (Crocker and Grier, 1996) to locate the position and size of each peak in the image above a threshold that yields extraction of a user-defined number of cells. The center is interpolated based on the brightness of all pixels in the bandpassed image within a user-defined region of radius R. The extent of interpolation R is independent of the bandpass thresholds, and should be chosen smaller than the upper bandpass limit, since features of size larger than the upper bandpass limit are suppressed in the filtered image. In this part, the user defines three parameters: lower bandpass (noise level in the image), higher bandpass (cell size), and the number of cells that are extracted in a sample frame.

#### Cell tracking algorithm

Once cell positions are determined in each frame, individual cells are mapped from frame to frame to enable unique identification of each cell. This is accomplished by mapping cells from one frame to the next in such a way that the cumulative square displacement between frames is a minimum value. Define A as the set of all one to one mappings from {1, …, n} to itself. Let a^*^ϵ *A* be the arrangement that minimizes the cumulative square displacement value, i.e.,:

$$a^{*}≜{\text{argmin}}_{a\;\epsilon \text{ A}}\left\{{\sum_{k=1}^{n}\left[x_{a(k)\;}\left(t+\delta t\right)-x_{k\;}\left(t\right)\right]}^{2}\right\},$$

where *x*_*k*_(δ) refers to the position of the k th cell at time δ. Observe that the above optimization is a bipartite minimum matching problem, which can be solved in polynomial time.

The tracking software can also be adjusted with a memory parameter to account for the possibility that some cells may not be visible enough to be located in a subset of the frames. Cells are then “remembered” for the subset of frames in which they are not visible. This mapping yields trajectories for each cell that could be mapped through the image sequence. The user defines three tracking parameters in this section. Apart from the memory parameter, the user sets the maximum frame to frame translation expected in the dataset and the number of frames in which a track should last to be valid.

#### Motion correction

From the shift in all measured trajectories in each pair of frames we then infer the overall shift and rotation of each frame. While the shift in the positions of all tracked neurons does not perfectly fit a rotation and translation (local distortions are also possible) we compute the rigid body transformation (translation and rotation) that minimizes residual displacements from one frame to the next using the Kabsch algorithm (Kabsch, 1976, 1978). These rigid body transformations are calculated for all frames to carry out motion correction for the whole image sequence.

Define $V\;=\;\left(x_{1,i\;},x_{2,i},\ldots ,x_{N,i}\right)\in {\mathbb{R}}^{2\times N}$ and *W* = (*x*_1, *f*_, *x*_2, *f*_, …, *x*_*N, f*_) ∈ ℝ^2 × *N*^ as position vectors for extracted cells where *i* represents the positions in the frame (source) and *f* the average positions we want to correct toward (target). Using the Kabsch's algorithm it can be easily observed that the optimal rotation matrix is:

$$R\;=\;{\left(S^{T}S\right)}^{\frac{1}{2}}\;S^{-1},$$

where *S* = *VW*^*T*^.

The Code is available through the github link: https://github.com/saghayee/Tracking-based-registration.

#### Validation of subpixel motion correction

**Figure 2:**
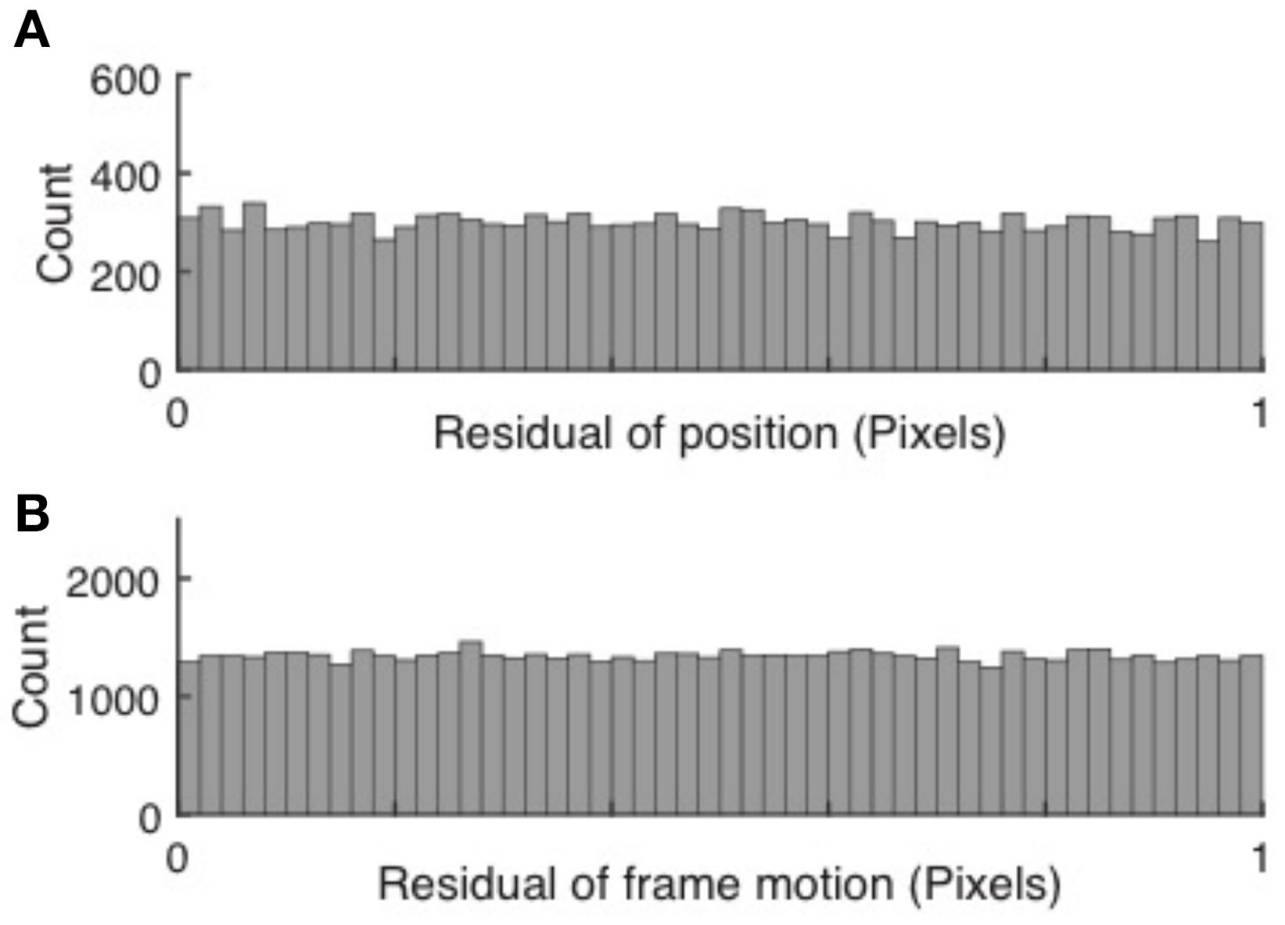
Validation of subpixel resolution particle finding and motion correction. **(A)** Distribution of the residual cell position after extraction for 67,148 cells extracted from 15,000 frames. **(B)** Distribution of the frame-to-frame residual displacement. The uniform distributions indicate sub-pixel resolution of both cell tracking and motion correction.

Subpixel precision can be achieved via peak finding, but only if each peak spans a sufficient number of bright pixels. If subpixel precision is achieved, the cell positions and subsequently the frame to frame motion, when measured in pixels, fall anywhere between integer pixel values with equal probability. Thus, the residuals have a random distribution between 0 and 1. Conversely, if peak finding and motion correction only achieved pixel resolution, the distributions would peak around both 0 and 1. Examples of uniform histograms for the residual values of both cell motion and frame jitter are shown in Figure 2.

## Results

We quantify brain motion using object-tracking methods adapted from the field of soft-matter physics outlined above. Fast, robust particle tracking algorithms have been first introduced two decades ago (Crocker and Grier, 1996) and refined extensively since then by others (Blair and Dufresne, 2008) and us (Losert et al., 2000). The particle tracking approach, described in the Methods section in detail, takes advantage of the fact that neuronal cell bodies are roughly spheroidal and resemble each other in size and shape. Our approach works best with a signal that is independent of neuronal activity and thus under ideal circumstances would appear at uniform brightness. This is achieved by transfecting neurons with constructs that besides supplying a Ca^2+^ indicator also label the nucleus or cytosol with activity independent label, e.g., mRuby2 or others (Peron S. P. et al., 2015; Rose et al., 2016). We benchmark our algorithm using image sequences obtained from the primary auditory cortex in awake mice using GCamp6s for Ca^2+^ and mRuby2 as a somatic marker (AAV-syn-mRuby2-GCaMP6s).

**Figure 3:**
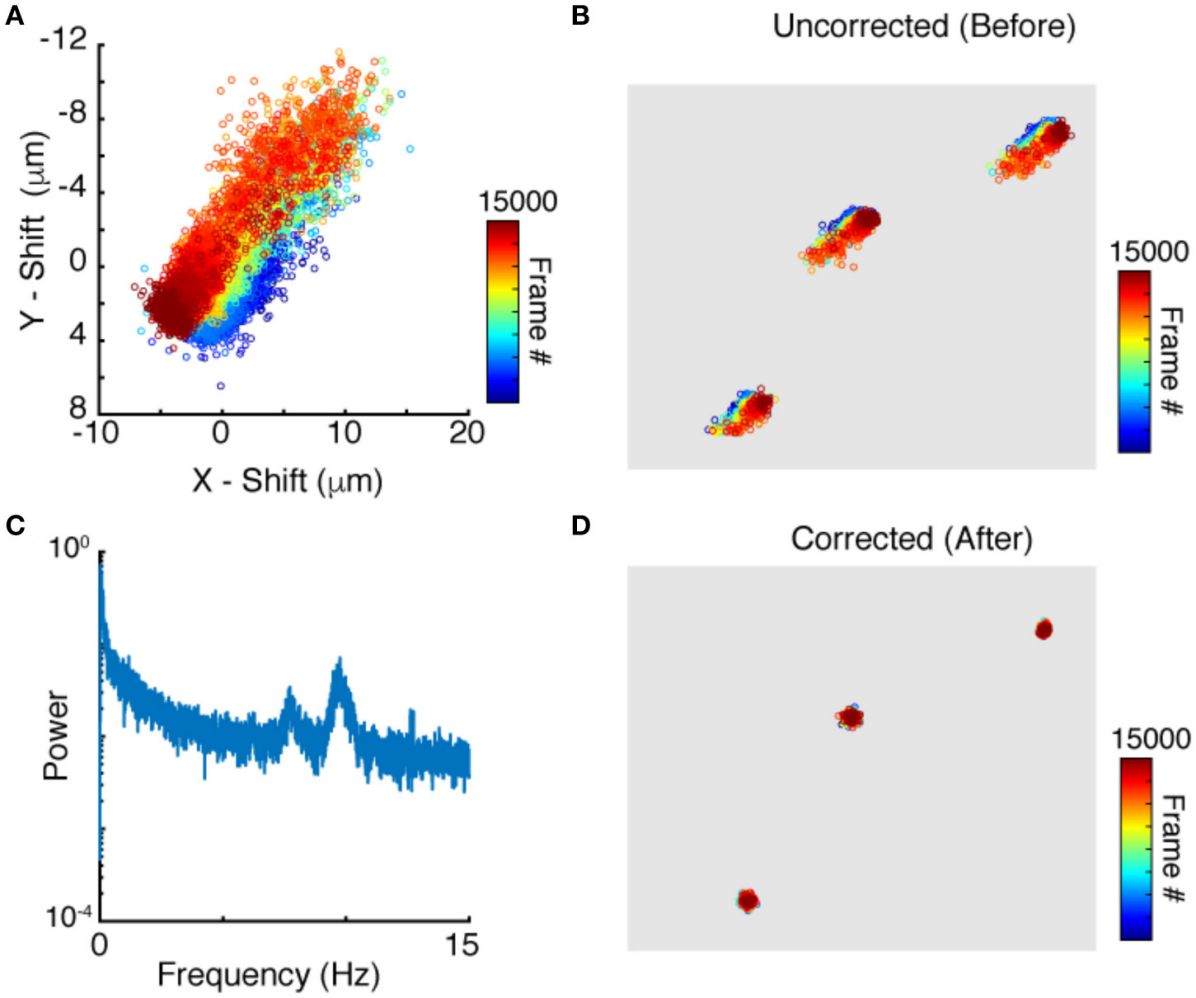
Particle tracking and residual cell motion. **(A)** Tracks of a single neuron in time. **(B)** Tracks for three neuronshighlight the similarities in trajectories. **(C)** Power spectrum of image jitter, **(D)** Tracks after motion correction.

Applying peak finding and particle tracking, detailed in the Methods section, yields the motion of each neuron independently. The time dependent position of a single neuron is shown in Figure 3A. Since tracks of nearby neurons look very similar (Figure 3B) it is reasonable to use the tracks for motion correction as described above. We compute the translational and rotational shift of an image from tracks of more than two points (*N* > 2). Two reference points, though sufficient in principle, are not enough given the uncertainty and errors in both imaging and processing. Since peak finding increases in accuracy with increased neuronal brightness, we use the brightest N tracked neurons for the motion correction algorithm outlined in the Methods section. To evaluate which frequencies contribute the most to the motion, the power spectrum of jitter is shown in Figure 3C. The power spectrum shows notable peaks on top of a regular noise spectrum at frequencies of 7. 7 and 9.7 Hz comparable to the expected heartbeat of a mouse. Correcting the position of each point for this jitter yields close to stationary points, with only very weak residual fluctuations, as shown in Figure 3D. We note that natural jitter can include deformations and rotations of the tissue. In the sample datasets, the algorithm detected rotations in the range of 0.6 degrees which corresponds to displacements of up to 3.6 μm in a 370 × 370 μm image.

When benchmarking for processing speed, our algorithm yields analysis of 500 images in 5.87 s or 85 frames per second on a six core 3.5 GHz Intel Xeon Mac with OSX and 64 Gb of RAM.

**Figure 4:**
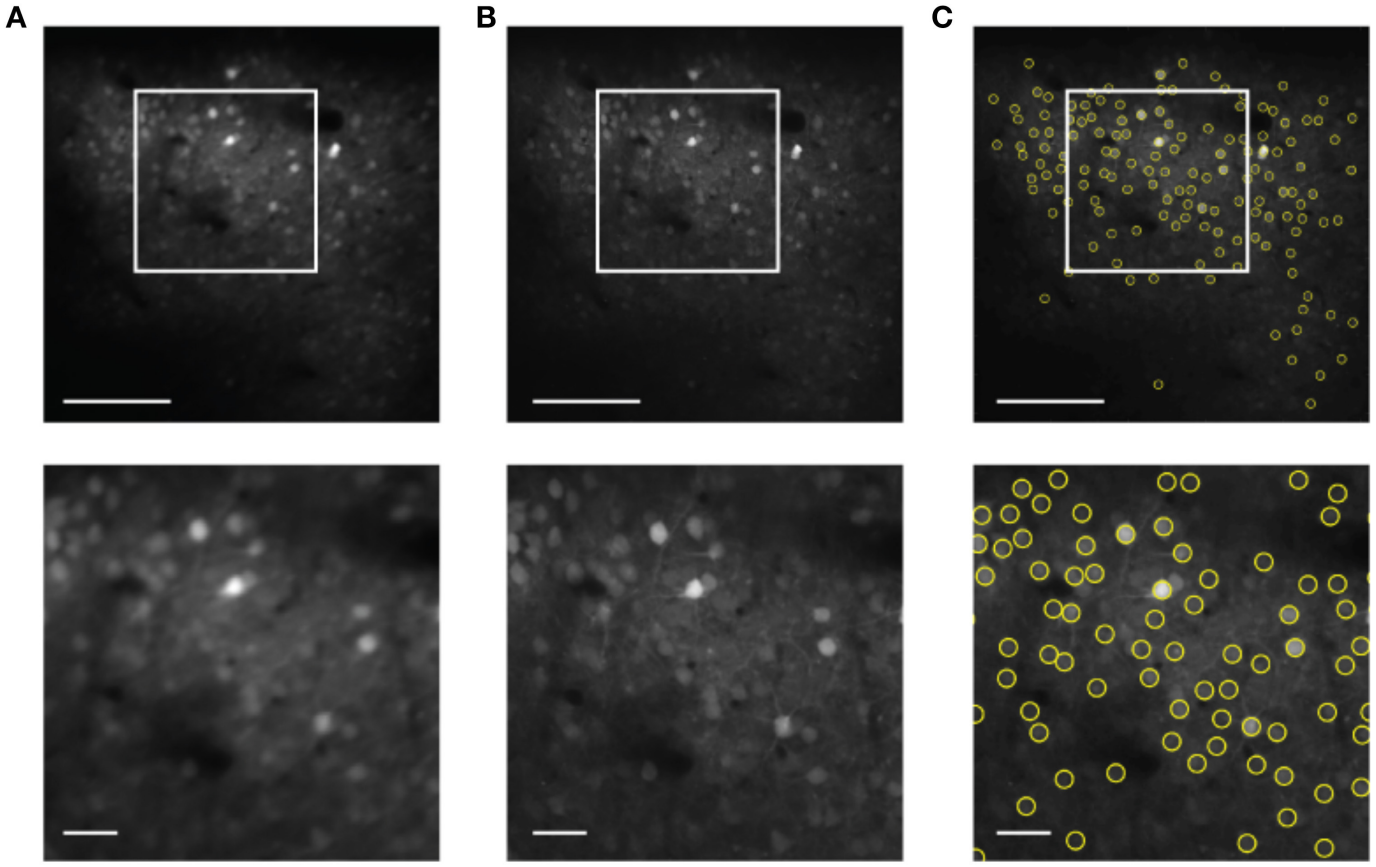
Cell finding from motion compensated averaged images in the static channel. The scale bars are 100 μm (top) and 20 μm (bottom). **(A)** Averaged image for the unregistered image sequence. **(B)** Averaged image after motion correction. **(C)** Automated cell finding. Yellow circles show the extracted cells.

The next step is to use the motion compensated images to identify neuronal ROI to be used for measurement of time traces of activity. Motion correction yields a sharp averaged image (Figure 4B) compared to the uncorrected average (Figure 4A). While a single frame is so noisy that peak finding only captures the brightest neurons accurately (Figure 1), peak finding on the averaged image reliably yields most of the cells visible by eye (Figure 4C). These brightness peaks provide automated input for established algorithms to identify ROI around each cell center, and to trace the image intensity in the ROI over time (Chen et al., 2013).

**Figure 5:**
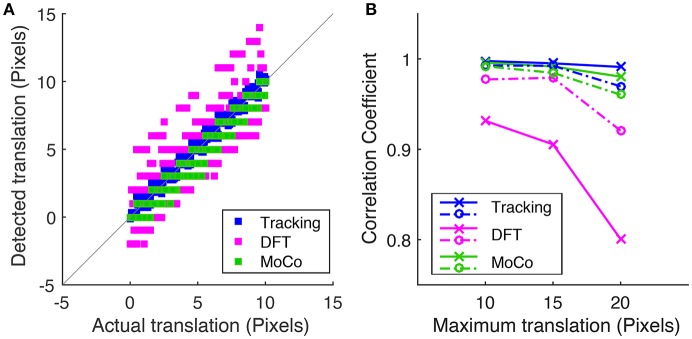
**(A)** Correlation of the detected frame to frame translation along the y-axis vs. the actual offset applied for tracking-based registration, DFT method and MoCo, where the corresponding simulated sequences were shifted by a maximum of 10 pixels. **(B)** Is a plot of the Pearson correlation coefficient between the measured translation and actual translation along the x-axis (dotted lines) and the y-axis (solid lines) vs. the maximum translational shift applied to the simulated sequence for each of the three algorithms.

To measure motion correction quality, we run the algorithm on a simulated data-set with known translational offsets and a known rotation of 1.2° in order to compare the tracking-based algorithm (blue) to the conventional DFT method (magenta) and MoCo algorithm (green). Figure 5A shows the correlation between the detected offset along the y-axis vs. the actual offset applied to each frame where the maximum translational shift applied to the sequence is 10 pixels. Figure 5B plots the Pearson correlation coefficient between the detected and actual translational offsets vs. the maximum translational offset applied to the image sequence for the three methods.

**Figure 6:**
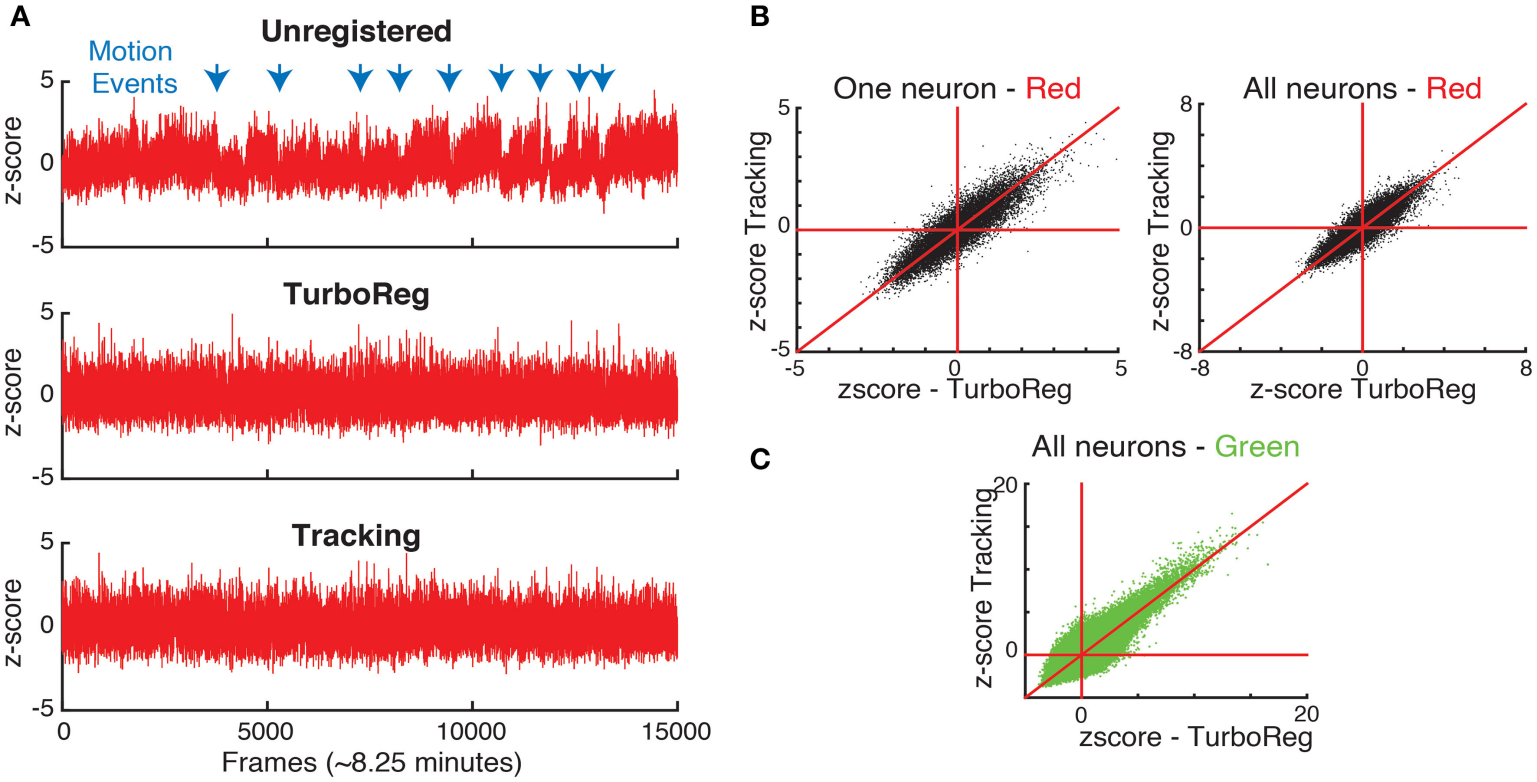
**(A)** Comparison of normalized intensities (z-scores) of the static channel vs. time for one representative neuron. **(B)** The z-scores obtained with tracking and TurboReg based motion correction match for one representative neuron, and all neurons. **(C)** Comparison of z-scores in the calcium imaging channel (green) for tracking and TurboReg corrected images.

Moreover, to show the effects of motion on the fluorescence signal we take advantage of the nominally uniform brightness of the second (in this case red) fluorophore with time, which we capture in a second, static channel. Therefore, the fluorescence intensity in each of the motion compensated ROI should be constant for the static channel. In fact, fluctuations in the static channel are indicative of both inaccuracies in motion correction and acquisition noise that is present in each individual frame as seen in Figure 1. We first measure the time sequence of these fluctuations using the z-score as a normalized measure (Kato et al., 2015). Figure 6 shows the z-score of one representative cell as a function of time for the original image sequence, after full image registration with TurboReg (Thevenaz et al., 1998), and after tracking-based motion correction. The unregistered image sequence has several sudden jumps in z-score indicative of sudden large changes in position, as well as slow drift in z-score indicative of slower shifts in the imaging location. Motion events (blue arrows) in particular may be misidentified as neuronal activity in spike inference algorithms. Both TurboReg and particle tracking based motion correction eliminate these artifacts. For all tracked neurons, the z-scores obtained with both motion correction methods are comparable for both the second control fluorophore (Figure 6B) and for the calcium signal (Figure 6C).

**Figure 7:**
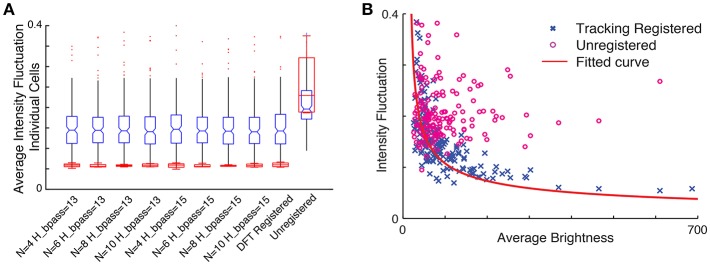
**(A)** Comparison of intensity fluctuations in the static channel for different tracking parameters with the DFT corrected sequence and unregistered stack. Blue, all cells; Red, four brightest cells. **(B)** Intensity fluctuation vs. average brightness of each cell in the static channel. The blue cross represents ROI extracted from the tracking registered stack while the circles in magenta are shows the ROI extracted from the unregistered stack. The red line shows the fitted curve to the registered ROIs ($\frac{1}{\sqrt{x-13.79}}$).

To assess how the quality of motion correction depends on tracking parameters and the number of tracked cells, we analyzed the same image sequence with different choices for parameter values, and compared the results with the DFT approach. For a robust baseline comparison to established practice, we used manual cell identification from the averaged images for all cases. Figure 7A shows the intensity fluctuation in the static channel measured from the four brightest cells (red) and for all 254 cells detectable in the averaged image (blue). The tracking-based analysis is robust to changes in parameters (for the number of neurons N between 4 and 10 and bandpass levels of 13–15 pixels, comparable to the size of a neuron), and comparable to the performance of DFT. Since the fluorescence from the static channel should be constant in time for each neuron, and at the same time the fluorophore uptake and thus fluorescence intensity levels vary significantly from neuron to neuron, we can determine whether fluctuations in measured intensity depend on the brightness of the neuron. Figure 7B shows that after motion correction, the fluctuations decrease with increasing brightness of the cell. For unregistered images, on the other hand, fluctuations are independent of brightness. The dependence on brightness approximately follows an inverse square root dependence on intensity, with a small intensity offset, as expected for a noisy system.

**Figure 8:**
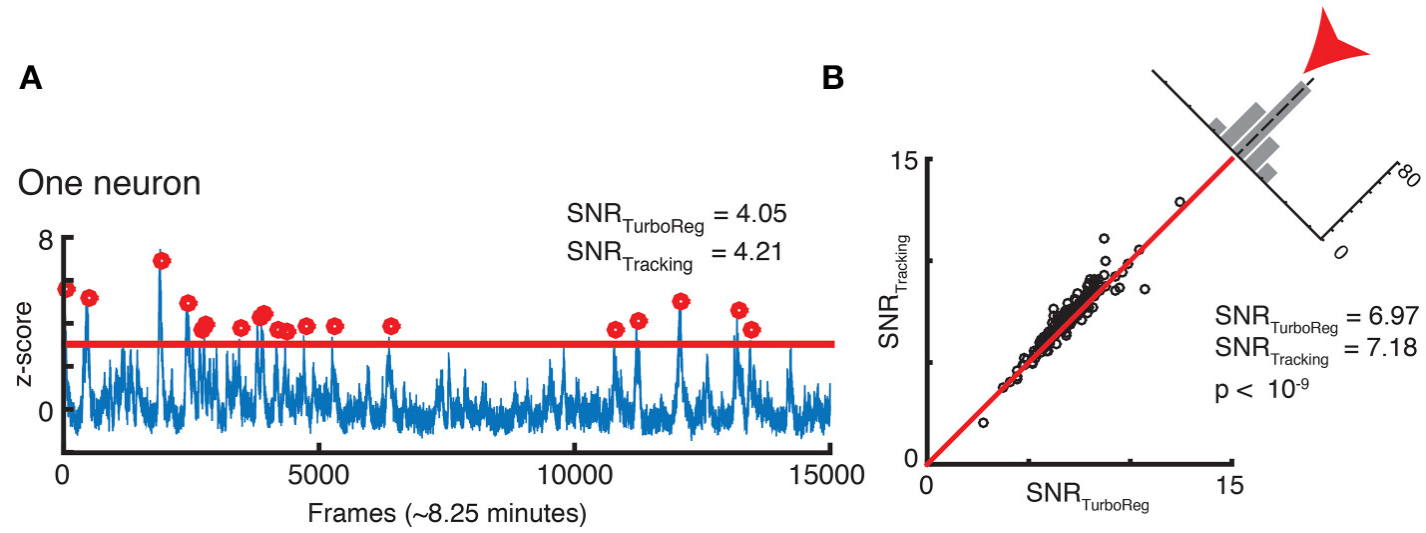
**(A)** SNR calculation for one neuron. **(B)** SNR correlation of tracking registration vs. TurboReg for all neurons in the FOV.

Finally, we assess whether our new tracking based motion correction yields a good SNR for the Ca^2+^ signal that represents neuronal activity fluctuations in the GCamp6 channel. The SNR is calculated as the mean of the signal (defined as the local maxima of the transient crossing a threshold at z-score of 3) divided by the standard deviation of the noise (defined as anything below this threshold). This measurement is particularly sensitive to the robustness of the algorithm, since the SNR depends on peaks in brightness above a threshold as shown in Figure 8A. We find very similar, if not slightly higher, SNR for each neuron with our tracking-based motion correction approach when compared to TurboReg. Figure 8B compares the SNR from all neurons in the tracking-based motion corrected sequence with that of TurboReg.

**Figure 9:**
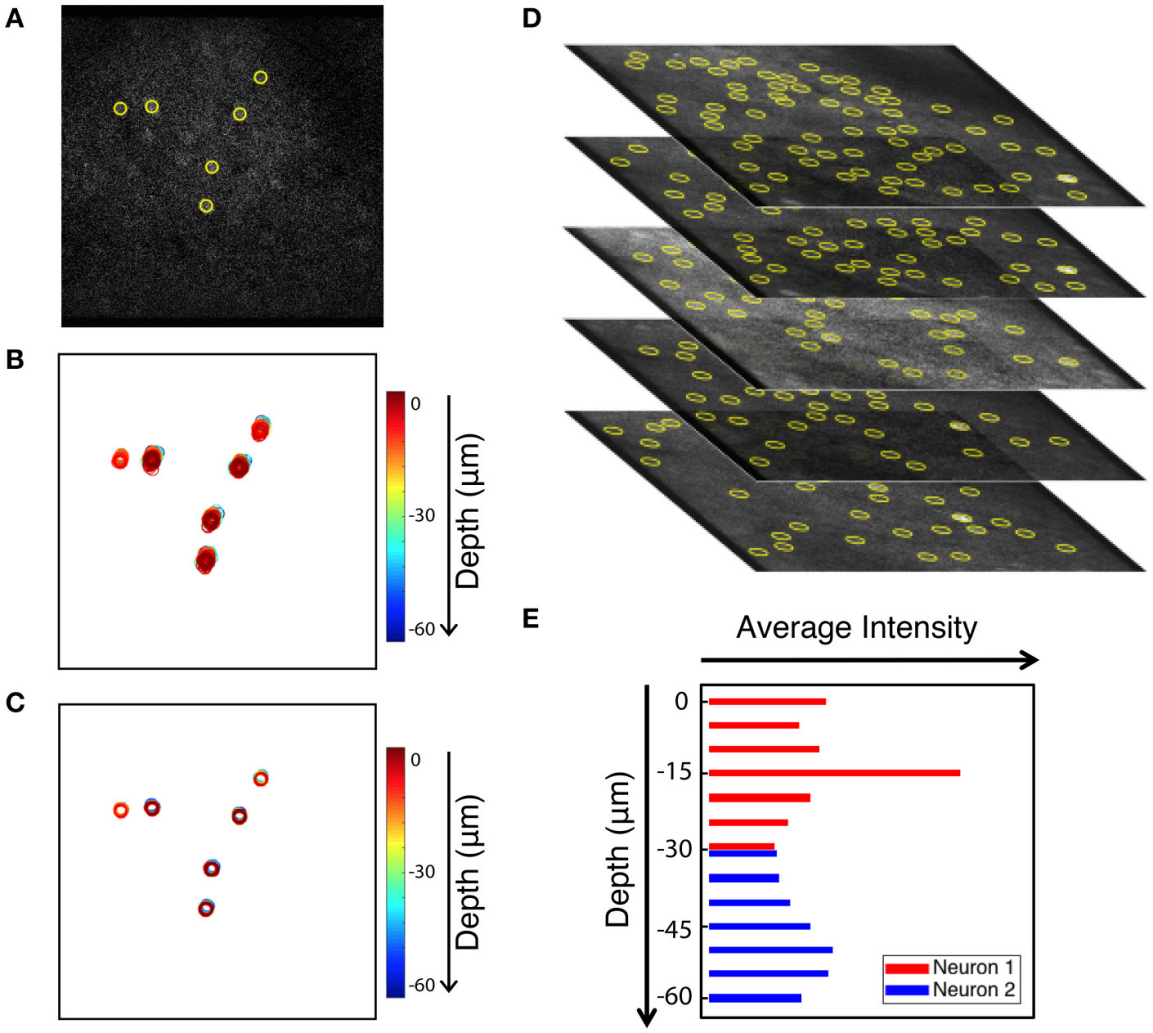
Extension of peak finding to three dimensions. Images are from the static channel. **(A)** Sample image with 6 extracted cells. **(B)** Overlay of the six cells identified in all layers at all times (unregistered). **(C)** Overlay of cell position after motion correction. **(D)** Brightest cells are extracted independently from each z-plane of the volume data image. Extracted cells are tracked through data acquisition timepoints for every plane. **(E)** Intensity of two sample cells in different Z planes. Yellow circles show the extracted cells.

As an added benefit, the tracking-based approach to motion correction also registers volume images. Volume imaging has become feasible due to the availability of faster scanning methods. Since Ca^2+^ signal has a time constant of 500 ms (Chen et al., 2013) it is now possible to record neuronal activity in several z-regions with a frame rate of 30 Hz without significantly diminishing acquisition concurrency. Volume imaging can allow for imaging of neurons in different layers, or to provide additional information about the same neurons. When applying our in-plane motion correction approach to image sequences, we shift each frame so neurons in the compensated image appear at the mean position when compared to all uncorrected images (i.e., we choose the mean image position as a reference position). For long enough image sequences, the mean positions of different z-planes coincide as we can see when identifying neuronal positions in multiple planes. Thus, correcting each plane to its mean position is sufficient to align a whole 3D volume. This is demonstrated in Figure 9. Six neurons that are extracted from a single frame are shown in Figure 9A. Figure 9B shows the uncorrected position overlays of six bright extracted neurons from different z-planes over time. Note that often the same neuron is picked-up by the algorithm in multiple neighboring z-planes. The corrected overlays are illustrated in Figure 9C after motion correction was carried out for each image plane independently. Motion correction to the average position yields a simple, yet powerful approach to create aligned 3D volume time lapse images. From such registered image stacks it is then possible to identify the average z-position of a neuron as well, based on the z-dependent fluorescence intensity of the ROI around a peak, as shown in Figures 9D,E. Thus, our approach can be extended to ultimately compensate for motion perpendicular to the imaging plane as well.

## Discussion

We introduce peak finding and particle tracking as a new motion correction approach that is fast and compatible with the low SNR nature of TPLSM neural activity data. It performs best with a time-invariant fluorescent signal generated with a secondary fluorophore. While having a second fluorophore adds experimental complexity, it not only facilitates accurate and fast motion correction but also yields independent fiduciary markers for each neuron. This allows for identification of all neurons independent of their activity levels. One alternative suitable for FRET based imaging is to add rather than subtract intensities of the two channels of a FRET probe, such as YC2.6 (Whitaker, 2010), which would yield an approximately constant total fluorescence intensity.

The tracking-based motion correction introduced in this paper does not rely on an averaged image for accuracy. Instead, it extracts the position of a few reliable bright cells in every frame, tracks them over time, and corrects for the displacement without using the averaged image as reference. Tracking only the brightest cells is both more reliable and faster computationally. Subpixel accuracy increases with the pixel size of a neuron, and does not require additional computations, such as up-sampling. Since each tracked cell is corrected to its mean position independently, the tracking method can be expanded for use in cases where distortions of the tissue become important, e.g., near blood vessels or when imaging very large areas. In addition, it can correct for rotations of the FOV that lead to displacements of 7 μm in a 370 × 370 μm as confirmed by simulations. While we only detect rotations of up to 0.6 degrees in the experiments when imaging an area of 370 by 370 microns, a comparable rotation would be very problematic when imaging at the mesoscale of several mm (Sofroniew et al., 2016; Stirman et al., 2016).

Our particle tracking based approach complements prior published work including whole image/region based registration algorithms, such as TurboReg or MoCo (Dubbs et al., 2016), and Fourier based methods, such as DFT registration (Guizar-Sicairos et al., 2008). Our approach has comparable accuracy, but as noted above yields subpixel resolution and rotation tracking without loss of performance. Since motion correction is based on measured displacements of individual cells, it will be possible to fit the measured displacements to image deformations as well. These advances in motion correction and spike inference methods should aid in improving data quality in imaging experiments (Harris et al., 2016), especially for mesoscale microscopy where image deformations and rotations are expected to become too large to neglect.

Peak finding has a second purpose in the workflow we introduce: Once image sequences are motion compensated (by any method) the peak finding algorithm is applied to the averaged images to determine the position of almost all cells visible to the eye in an unbiased way. Having a reference signal that is time invariant allows for calibration of the SNR and aids in robust peak finding. Conversely, the subpixel accuracy motion correction—and the ability to adjust for local deformations of the image field if needed based on the independent track of each neuron—is a strong basis for advanced spike inference and analysis tools. A novel phase correlation based method for example, assumes motion correction (Pnevmatikakis and Giovannucci, 2017).

Since the analysis yields in-plane motion correction that is registered across planes for three-dimensional image sequences, our approach provides a basis for motion correction in all three dimensions, which promises to further enhance the signal to noise and thus enable even more accurate determination of the neuronal code.

## Ethics statement

This study was carried out in accordance with the recommendations of University of Maryland Institutional Animal Care and Use Committee. The protocol was approved by the University of Maryland Institutional Animal Care and Use Committee.

## Author contributions

SA led analysis effort and drafted the manuscript. DW carried out experiments and contributed to analysis. ZB contributed to data analysis. EM contributed to initial analysis workflow. MH contributed to workflow design and analysis, PK and WL designed the experiment and analysis workflow and co-wrote the manuscript.

### Conflict of interest statement

The authors declare that the research was conducted in the absence of any commercial or financial relationships that could be construed as a potential conflict of interest.
